# Diet, lifestyle factors and hypercholesterolemia in elderly men and women from Cyprus

**DOI:** 10.1186/1476-511X-9-107

**Published:** 2010-09-24

**Authors:** Evangelos Polychronopoulos, Demosthenes B Panagiotakos, Anna Polystipioti

**Affiliations:** 1 Department of Dietetics - Nutrition, Harokopio University, Athens, Greece

## 

It is clarified that the food frequency questionnaire (FFQ) used in the article published in Lipids in Health and Disease [1], as well as in the whole MEDIS study, was a short, semi-quantitative FFQ with 15 questions regarding the basic food groups consumed, as well as with some additional questions regarding beverages' drinking and other nutritional habits [2-4]. The reference Katsouyanni et al., [5] was cited in order to support that in epidemiologic research is common procedure measuring nutritional habits as an average per week during the preceding year (even in the elderly), and was not referring to the FFQ used.
